# Analyses of the Microbial Diversity across the Human Microbiome

**DOI:** 10.1371/journal.pone.0032118

**Published:** 2012-06-13

**Authors:** Kelvin Li, Monika Bihan, Shibu Yooseph, Barbara A. Methé

**Affiliations:** J Craig Venter Institute, Rockville, Maryland, United States of America; Virginia Commonwealth University, United States of America

## Abstract

Analysis of human body microbial diversity is fundamental to understanding community structure, biology and ecology. The National Institutes of Health Human Microbiome Project (HMP) has provided an unprecedented opportunity to examine microbial diversity within and across body habitats and individuals through pyrosequencing-based profiling of 16 S rRNA gene sequences (16 S) from habits of the oral, skin, distal gut, and vaginal body regions from over 200 healthy individuals enabling the application of statistical techniques. In this study, two approaches were applied to elucidate the nature and extent of human microbiome diversity. First, bootstrap and parametric curve fitting techniques were evaluated to estimate the maximum number of unique taxa, S_max_, and taxa discovery rate for habitats across individuals. Next, our results demonstrated that the variation of diversity within low abundant taxa across habitats and individuals was not sufficiently quantified with standard ecological diversity indices. This impact from low abundant taxa motivated us to introduce a novel rank-based diversity measure, the Tail statistic, (“τ”), based on the standard deviation of the rank abundance curve if made symmetric by reflection around the most abundant taxon. Due to τ’s greater sensitivity to low abundant taxa, its application to diversity estimation of taxonomic units using taxonomic dependent and independent methods revealed a greater range of values recovered between individuals versus body habitats, and different patterns of diversity within habitats. The greatest range of τ values within and across individuals was found in stool, which also exhibited the most undiscovered taxa. Oral and skin habitats revealed variable diversity patterns, while vaginal habitats were consistently the least diverse. Collectively, these results demonstrate the importance, and motivate the introduction, of several visualization and analysis methods tuned specifically for next-generation sequence data, further revealing that low abundant taxa serve as an important reservoir of genetic diversity in the human microbiome.

## Introduction

The human body is host to microbial communities (microbiome) whose abundances are estimated to exceed the number of human cells by at least an order of magnitude [1]. These communities are thought to influence human physiology through processes related to development, nutrition, immunity, and resistance to pathogens [2], [3], [4], [5], [6]. The HMP [7] was initiated to probe the nature and extent of the microbial communities living in and on the human body of so called “normal” adult donors in an effort to better understand their role in human health and disease, thus providing a critical baseline for future metagenomic studies of the human microbiome.

Since the vast majority of microbes are as yet uncultured by current techniques [8], molecular-based, culture-independent techniques, such as the use of 16 S profiling have provided important new insights into the diversity of the microbial world across a variety of environments [9], [10], [11], [12], [13] including the human microbiome [14], [15], [16], [17]. The HMP has generated an unprecedented scale of 16 S profiles to investigate the microbial diversity of the human microbiome consisting of over 230 donors and up to 18 body habitats across the oral cavity, skin, distal gut (stool), and when applicable, vaginal body regions (Table 1).

**Table 1:** Diversity indices computed on all genera-based taxonomic units.

Location	Shannon Entropy				Tail				S_max_			N
	Median	Lower	Upper	Pooled	Median	Lower	Upper	Pooled	Median	Lower	Upper	
**Oral**												
Buccal mucosa	1.664	0.783	2.541	1.999	4.324	1.485	7.953	7.738	315	172	6,592	201
Hard palate	2.098	1.068	2.695	2.338	5.747	2.911	8.422	9.344	396	202	15,442	199
Keratinized gingiva	1.588	0.463	2.495	1.938	2.973	0.818	6.914	5.907	478	146	11,910	208
Palatine tonsils	2.412	1.476	2.810	2.792	6.389	3.606	9.432	9.897	237	179	9,474	207
Saliva	2.655	1.957	3.008	2.935	8.329	4.875	11.312	11.587	332	199	7,406	183
Subgingival plaque	2.634	1.948	3.049	3.092	7.730	4.498	10.843	11.754	568	177	10,366	206
Supragingival plaque	2.589	1.803	3.002	2.958	7.026	3.560	10.227	9.894	502	155	20,313	205
Throat	2.420	1.261	2.888	2.793	6.586	3.709	9.955	11.269	389	228	13,111	198
Tongue dorsum	2.304	1.552	2.755	2.669	5.466	3.110	7.983	7.687	415	128	10,561	205
**Skin**												
Anterior nares	1.498	0.527	2.418	2.264	3.383	1.331	7.378	12.753	609	374	7,730	173
L Antecubital fossa	2.149	0.499	3.318	2.797	8.882	2.451	24.566	31.345	501	367	15,534	89
L Retroauricular crease	0.843	0.176	2.351	1.277	2.106	0.549	8.418	10.540	504	315	5,808	193
R Antecubital fossa	1.985	0.506	3.279	2.650	8.852	2.471	22.828	29.603	432	352	2,507	94
R Retroauricular crease	0.887	0.115	2.282	1.405	2.030	0.488	9.675	11.761	486	323	21,188	199
**Vaginal**												
Mid vagina	0.211	0.021	1.680	0.696	0.911	0.124	4.184	5.258	176	141	3,823	95
Posterior fornix	0.071	0.011	1.638	0.467	0.398	0.066	2.972	3.237	154	112	3,530	95
Vaginal introitus	0.423	0.045	1.996	1.003	1.514	0.216	4.660	6.967	170	134	8,310	86
**Stool**	1.663	0.412	2.615	2.062	4.137	1.050	7.231	8.513	226	154	8,504	208

Understanding microbial community diversity is a critical first component for studying the human microbiome in order to elucidate the distribution and assembly patterns of microbial communities across body habitats and individuals and to facilitate microbial ecological and biological studies [15], [18], [19]. Characterization of microbial community diversity from 16 S sequence reads requires three basic steps. First, reads must be classified into distinct taxonomic units thus creating a taxonomic profile. Next, the taxonomic profile is assessed by quantifying richness, or number of taxonomic units present, and finally the evenness, based on the abundance of taxonomic units [20]. Commonly, such profiles are generated using two general approaches based on the sequences acquired through each 16 S rDNA sample.

The first method is based on the assignment of sequences to a taxonomic hierarchy [21]. For example, the Ribosomal Database Project (RDP) Classifier, uses a naïve Bayesian classifier to rapidly classify bacterial 16 S rRNA sequences into a higher-order taxonomy (Bergey’s Taxonomic Outline of the Prokaryotes [22]). Since a reference sequence from the database is utilized, sequences with minor sequencing errors may still be properly associated. However, novel organisms that have not had their 16 S sequences included into the database may be misclassified or considered unknown.

The second approach is independent of taxonomic classification and uses sequence similarity to form clusters within a predefined percent similarity, for example, 97% similarity [21]. All reads within each cluster are considered to be in the same operational taxonomic unit, or “OTU.” OTU-based taxonomic profiles are prone to under-clustering. This occurs when reads from the same organism are divided into multiple taxonomic units due to sequencing error, or when there is significant diversity of the 16 S copies within a single organism [23], [24]. Since OTU-based taxonomic profiling is database independent, two benefits of its utilization include potentially differentiating between strains of the same species, and generating OTUs for as yet uncharacterized organisms. Due to the shortcomings and advantages of each approach, both techniques offer alternative views of a sample’s taxonomic profile, thus being both complementary and confirmatory. When the combined approaches are compared, resulting inconsistent inferences may elucidate interesting aspects of the samples being analyzed.

After generation of the 16 S profile, the inventory of the microbial community within a sample is assessed by quantifying the richness (defined as the number of unique taxa present), the diversity (which combines the concepts of both richness and evenness), or the abundance of taxonomic units that have been accounted for by the taxonomic profile [21]. Diversity indices are often calculated in community diversity studies as they represent the distillation of this information into a single positive real ℝ number [25] whose magnitude can be more easily compared.

Estimating the maximum richness S_max_, in microbial community environments, is an area of ecological and biological interest. This information can also be used in a practical sense to generate sequencing coverage estimates and to determine the proportion of the microbial community that has been discovered as the sequencing effort is progressing [26]. A commonly used visualization tool to represent the discovery rate of new taxonomic units as samples are taken is the rarefaction curve. The x-axis of the graph is labelled with the number of samples (or donors) that have been observed, and the y-axis is the number of unique taxonomic units that have been observed within the samples collected thus far. The instantaneous slope and height of the curve inform the analyst about both the expected discovery rate of new taxonomic units and how many taxonomic units have been discovered at a particular point. S_max_ is found at the height of the curve when the slope is zero, i.e., the sampling saturation point. In previous studies, due at least in part to the prohibitive cost of sampling and sequencing, rarefaction curves based on empirical data have not typically acquired a large number of samples. Therefore, for communities with great diversity, the instantaneous slope of the last point of sampling does not become zero, as not all taxonomic units have been observed. Thus, to predict the number of taxonomic units in the community based on a rarefaction curve that is still climbing, the use of extrapolation is a reasonable next step. This is a different approach than the estimation of S_max_ based on the combined distribution of taxa across all samples for a specific body site. In this latter scenario, parametric finite mixture models or non-parametric coverage-based estimates, as implemented in Catchall [27], would be applicable. Efforts have been made to extrapolate the rarefaction curve with various parametric models, such as the poisson log-normal cumulative distribution function (CDF) [28]. In general, parametric statistical techniques can produce more accurate, precise and robust estimates, with greater statistical power than nonparametric methods, provided that the model fits the data correctly. When these assumptions are valid, estimating constants for the underlying distribution functions can be performed with optimization algorithms. However, when the model is only approximate, sparse sampling compounds the unreliability of parametric curve fitting, resulting in overfitting and outcomes that are poorly predictive. As a result, these techniques have not been generally recommended. However, the scope of the HMP has provided the community with a large number of samples from a large set of donors and a broad range of body habitats, enabling the realistic exploration of parametric modelling techniques. In particular, for each body habitat rarefaction curve, values for S_max_ were calculated and compared by applying these curve fitting approaches. Four well-understood CDFs, log-normal, gamma, Pareto [29], and Fréchet [30] were chosen for consideration in part as they are suitable for addressing ecological distributions that may be highly skewed towards the most dominant taxa.

The difficulty of measuring the microbial diversity in a sample is compressing the complexity of a profile, with a multi-dimensional representation of taxonomic abundance, into a single scalar statistic. At the same time, the number of reads per sample for a metagenomic sample is unlikely to have been sufficient to sample every organism, especially when a collected sample contains a large number of low abundant organisms, i.e., the “rare biosphere” [9]. Commonly used ecological diversity indices for quantifying intrasample diversity, include the Shannon index, a measurement of entropy and the uncertainty of the sampling outcome, and Simpson’s diversity index, a description of the probability that randomly drawing two reads from a sample will produce the same taxon. In terms of application to ecological studies, each of these indices was originally derived, or adapted from macroecology. As such, individually they can perform well when approximating the microbial diversity of common taxa, however each may fall short as a single complete measure when examining the numerous low abundant organisms that dominate the composition of many microbial communities.

**Figure 1:**
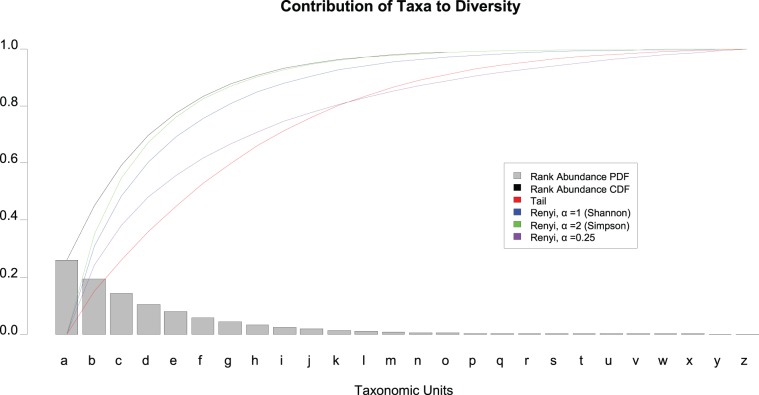
Contribution of taxa to diversity. A theoretical rank abundance curve (a PDF) is overlayed with its CDF (black) as a “Pareto chart”. The overlaid colored lines represent each diversity index as lower abundant taxonomic units are included. For example at “c”, the height of each curve represents the relative value of the index if the sample were only composed of a, b, and c. The more quickly an index curve reaches it maximum normalized value of 1.0, the less the index is capable of resolving low abundance taxonomic units. From the graph, it can be observed that the Shannon and Simpson diversity indices approach their saturation point more quickly than the Tail statistic or a Renyi entropy with a fractional alpha.

Both the Shannon and Simpson diversity indices have been shown by Hill [31] through Rényi’s definition of generalized entropy [32] to have similar characteristics, but differing only in the contribution of low abundant taxonomic units to the magnitude of the calculated statistic. Rényi unified the Shannon and Simpson diversity indices as entropies with a parameter α, the power to which the contribution of taxonomic abundances are raised. α’s of 2, 1, and 0, are associated with Simpson’s index, Shannon’s index, and the total number of species detected, respectively. It is possible to utilize fractional α’s, eg. 0.25, to increase the contribution of the low abundant taxonomic units to a desired impact (Figure 1), but if their specification is arbitrary, resultant index values may not have meaningful probabilistic or information theoretic interpretations.

The estimated S_max_ is dependent on the number of taxonomic units and their relative abundance across all samples from the site of interest. Thus it may be expected that a strong correlation exists between calculated diversity indices such as the Shannon index and S_max_ between all samples. However, our investigations suggest that this index was unable to capture enough of the low abundant taxa to correlate well with the estimated S_max_ for many body habitats. This finding motivated us to formulate the Tail statistic, τ, a rank-based diversity measure with a much stronger correlation to S_max_, and with intuitive characteristics matching the well-understood standard deviation statistic. As described more rigorously under Materials and Methods, τ is the standard deviation of the rank abundance curve had it been made symmetric by reflection around the most abundant, or first, taxon. The more concentrated the taxa are to a few members, the smaller τ becomes. The sensitivity of τ to low abundant taxonomic units is comparable to a Rényi entropy with fractional α, but with a probabilistic interpretation. τ consists of the number of taxonomic units as a unit of measure and due to its similarity to the standard deviation statistic, σ, further takes advantage of existing known properties of σ such as the Chebychev’s inequality [33]. The τ statistic provides an important complement to the study of microbial diversity as it has been derived to suit the nature of 16 S profiles which tend to exhibit a long-tailed distribution. These distributions reflect the nature of species abundance in which many sequences reside in a few taxonomic units while the majority of taxa are represented by only a few sequences. As such, τ more accurately captures the contribution of low abundant taxa facilitating an improved ability to elucidate the nature and extent of variation within and between individuals and body habitats over time, a central topic of study in human microbiome research [34].

In this study, we present results of an investigation of human microbiome diversity across habitats and individuals from 16 S profiles generated by the HMP. First, we present the application of parametric curve fitting techniques to estimate the maximum number of taxa and the instantaneous discovery rates in human body habitats. Next, we present comparisons of the Shannon, τ and S_max_ diversity estimates between OTU and taxonomy (genera)-based taxonomic units, and between common and all taxonomic units. Further, by the introduction of new statistical analyses tuned for next generation sequencing and improved methods to visualize and interpret their output, we not only provide a better understanding of the diversity across body sites and individuals, particularly through the contribution of low abundant taxa, but we also show how various approaches to diversity analyses complement and confirm these observations.

## Results and Discussion

The Shannon diversity index and τ statistic were calculated for each body habitat and the median and 95% confidence intervals (CIs) were recorded in Tables 1–2. The confidence intervals around the medians highlight the variability of diversity among individuals across body habitats. To look at the pooled diversity for each habitat across all individuals, the same statistics were calculated on a taxonomic distribution generated by combining the taxonomic distributions equal-weighted by individuals. The values for the median and 95% confidence intervals for S_max_ were generated with bootstrapping and then CDF curve fitting.

**Table 2:** Diversity indices computed on all OTU-based taxonomic units.

Location	Shannon Entropy				Tail				S_max_			N
	Median	Lower	Upper	Pooled	Median	Lower	Upper	Pooled	Median	Lower	Upper	
**Oral**												
Buccal mucosa	1.903	0.709	3.039	2.434	7.406	2.344	16.858	36.236	1,238	875	16,795	201
Hard palate	2.432	1.390	3.160	2.816	9.215	4.047	17.322	34.390	2,171	853	15,848	199
Keratinized gingiva	1.721	0.810	2.811	2.264	3.687	1.051	11.490	25.561	945	654	13,223	208
Palatine tonsils	2.877	1.697	3.448	3.534	12.535	5.298	23.037	79.686	3,958	1,529	27,400	207
Saliva	3.143	2.498	3.682	3.599	16.276	7.829	27.130	67.358	2,673	1,437	23,728	183
Subgingival plaque	3.175	2.231	3.676	3.877	16.444	6.219	28.829	91.758	4,395	1,792	30,450	206
Supragingival plaque	3.005	1.935	3.615	3.575	13.258	5.331	24.574	75.823	6,111	1,581	27,038	205
Throat	2.830	1.349	3.370	3.408	11.485	5.425	20.861	53.181	1,570	1,158	22,531	198
Tongue dorsum	2.609	1.786	3.216	3.109	9.518	4.771	17.449	66.752	2,178	1,530	25,280	205
**Skin**												
Anterior nares	1.656	0.762	2.694	2.532	4.583	1.715	14.190	46.318	1,350	894	17,931	173
L Antecubital fossa	2.496	0.429	3.738	3.219	11.847	3.086	49.009	107.121	1,477	1,039	25,517	89
L Retroauricular crease	0.855	0.177	2.822	1.335	2.708	0.610	15.905	35.921	1,285	800	16,752	193
R Antecubital fossa	2.143	0.487	3.746	2.982	12.340	3.185	48.603	100.726	1,337	1,031	21,389	94
R Retroauricular crease	0.873	0.117	2.421	1.501	2.450	0.583	15.601	49.117	1,410	890	23,636	199
**Vaginal**												
Mid vagina	0.219	0.016	2.072	0.782	1.201	0.113	8.058	16.459	487	362	8,610	95
Posterior fornix	0.086	0.003	1.911	0.549	0.420	0.018	4.476	10.112	335	223	6,134	95
Vaginal introitus	0.433	0.034	2.722	1.242	1.914	0.207	12.179	25.278	635	426	10,601	86
**Stool**	2.583	1.072	3.849	3.561	17.259	2.944	70.542	418.358	7,010	5,414	86,652	208

### The Diversity Across Body Habitats

**Figure 2:**
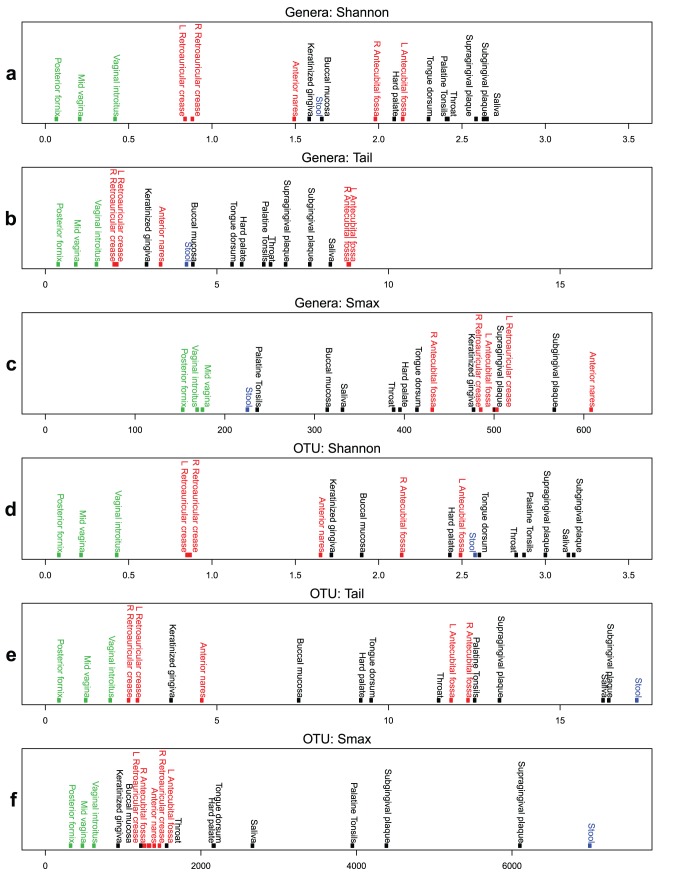
Body habitats ordered by diversity measure. Body regions are color coded, Oral-black, Skin-red, Vaginal-green, and Stool-blue. Subfigures a, b, and c, were computed on genera-based taxonomic units. Subfigures d, e, and f, were computed on OTU-based taxonomic units.

An examination of genera and OTU-based taxonomic units yielded largely similar results. The vaginal region had the lowest diversity, while the skin and oral regions were largely overlapping. Using the one-sided Wilcoxon Rank Sum Test (WRST) to test for differences in the medians, oral and skin regions contained more diversity than the vaginal region (oral versus vaginal, p-value = 0.0045, skin versus vaginal, p-value  = 0.0179) (Tables 1 and 2, Tables S1a–f ). The relative diversity of the stool samples fell among the oral and skin regions using the Shannon diversity measure, but appeared to have the greatest diversity based on τ for OTUs (Figure 2).

Although the skin and oral regions largely overlapped in diversity, the WRST for genera and OTUs indicated that the oral region had more diversity than skin using the Shannon diversity measure (p-value  = 0.0095). However, using the τ statistic, the oral region was not significantly more diverse than skin for both genera and OTUs with α = 0.05. These body region differences suggest that skin tended to possess a significant degree of low abundant taxa that the Shannon diversity was unable to capture. Left and right measurements were similar in both the retroauricular crease and antecubital fossa. These symmetrically sampled body sites provided a useful measure of the amount of variation that can be expected within an individual.

These results highlight how the τ statistic’s assessment of low abundant diversity could result in differences in the assertions made of relative microbial diversity when comparing body regions. Thus, methodologies which help to better elucidate the subtle variations of microbiome diversity are critical in understanding how body regions support alternative microbial communities.

An examination of the 95% confidence intervals across all body habitats reveals that the magnitude of variance of diversity observed between individuals is often greater than the variability of diversity at a specific body habitat across individuals. For example, the genera-based median Shannon diversity ranged from 0.071 (posterior fornix) to 2.655 (saliva) and the 95% CIs for the left antecubital fossa ranged from 0.499 to 3.318. τ statistic median values ranged from 0.398 (posterior fornix) to 8.882 (left antecubital fossa), with the 95% CI for the left antecubital fossa ranging from 2.451 to 24.566. The wide variation of both the Shannon and τ diversity measures for the left antecubital fossa suggests that diversity in these body habitats varied significantly for both the common and rare taxa.

### The Diversity Across Individuals

The diversity across individuals for a specific body habitat cannot be understood by looking at the median and 95% CIs across individuals, alone. Since the diversity within a body habitat or region is based on the number of taxonomic units that the environment can support, these numbers cannot differentiate whether the few (or many organisms) a site can support in one individual, are the same organisms in another individual. As a result, a set of individuals with low diversity at a habitat of interest when combined as a group of individuals, may sustain a much larger group of organisms, however mutually exclusive. To capture the taxonomic profile of a group of individuals, a pooled taxonomic profile was constructed by combining, in equal-weight, the taxonomic profiles of all individuals for a specific habitat. The diversity statistics were then computed on these combined (pooled) profiles.

**Figure 3:**
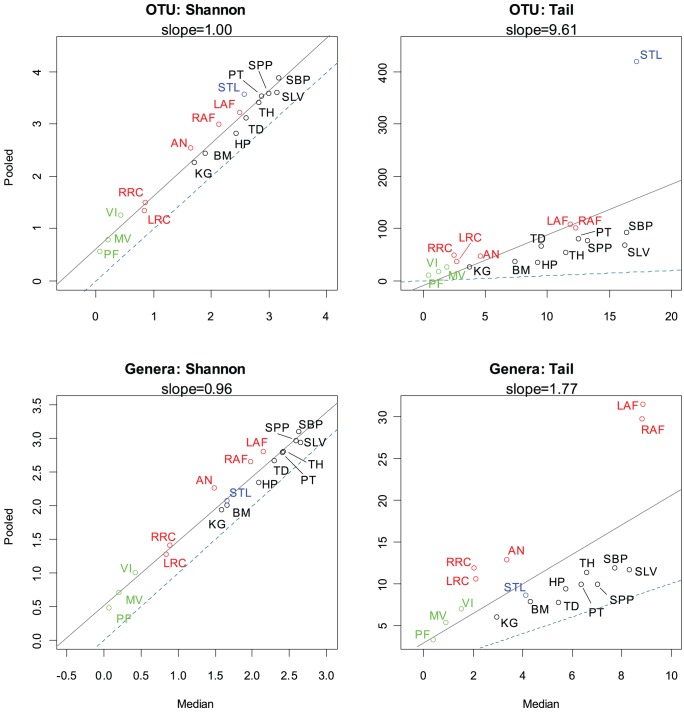
Comparison of diversity indices for median versus pooled taxonomic profiles. Simple regression lines were drawn in solid black for each median individual versus pooled samples scatterplot. The dashed blue lines (slope  = 1, y-intercept  = 0) represent where a hypothetical (median  =  pooled) relationship would exist if all individuals had identical taxonomic profiles. Both the OTU-based and genera-based comparisons using the Shannon diversity index indicate only a slight and almost constant elevation of the diversity between the median individual and pooled samples. However, τ is able to capture the lengthening tail attributed to the low abundant taxa that are exclusive to certain individuals. See Table S2 for a mapping of abbreviations to habitat names. Green, red, black and blue points represent vaginal, skin, oral, and stool body regions, respectively.

A comparison of the median diversity versus the pooled diversity can be used to elucidate the extent to which habitats have mutually exclusive diversity between individuals. This mutually exclusive diversity then represents organisms that are found in a subset of the population but not in the remainder, and vice versa. Generally, when the taxonomic distributions of two similar sites are combined, the measured diversity should not increase significantly. However, if new taxa are introduced or evenness in the distribution is increased, then the diversity of the combined distribution will be greater than the diversity measured individually. Since much of the mutually exclusive diversity tends to be found in the low abundant fraction when the equal-weighted pooling is performed, the Shannon diversity index does not differentiate well between the median and pooled diversity. A scatterplot of median individual diversity versus pooled diversity was generated for both the Shannon diversity index and τ on both OTU- and genera-based taxonomic units (Figure 3 and Table S2 for abbreviation to sample mapping). A simple linear regression line was drawn to indicate the mean ratio between pooled and individual diversity. If all individuals held identical taxonomic distributions, the regression line would have a slope of 1 and y-intercept of 0, since there would be no difference between a single median individual when compared to the collective taxa across all individuals.

For OTU- and genera based taxonomic units, slopes of 1.01 and 0.967 were observed respectively, using the Shannon diversity measure, misleadingly suggesting that there is little increase of diversity between the median individual and a pooled collection of samples from the population. However, a look at the τ statistic reveals significantly different outcomes. The slope of the regression line for the τ statistic is 9.617 and 1.768 for OTU- and genera-based taxonomic units, respectively. In particular, for OTUs it can be seen that the stool samples consist of significantly more mutually exclusive diversity than other body habitats. For genera-based taxa, the left and right antecubital fossae have the greatest mutually exclusive diversity. The pooled-to-median ratios for both OTU and genera-based taxonomic units reveal that the skin samples have more mutually exclusive diversity than the oral samples. This suggests that the taxonomic diversity in human oral habitats is shared more closely in the human population than skin habitats. The vaginal samples, because of their extremely low diversity, did not exhibit significant mutually exclusive diversity between donors, likely due to the unusual selective pressure of the environment. This analysis reveals that systematic differences between microbial communities do exist, however their detection must be facilitated with the proper statistical measures, such as the τ statistic, that are sensitive to low abundant taxonomies.

### Defining a Core Microbiome Across Individuals

Determining the ubiquity of taxa across body habitats is a central component to the study of microbial community diversity in healthy or normal individuals. In this study, we have shown that there is high variation in diversity between individuals at the same body habitats, a finding which confounds the determination of common or “core” taxa across sets of individuals. While diversity contributed by low abundant microorganisms will no doubt exist in all body habitats, our analysis of pooled versus individual diversity using the τ statistic indicates that the composition of this low abundant component will not necessarily be identical among individuals. A naïve measurement of taxonomic ubiquity will not take into account the significance of low abundant taxa, thus producing an oversimplified “presence or absence” determination of microbiome composition. A more useful model for addressing the conservation of taxonomic abundance across a set of samples, in our case various individuals, must be probabilistic and conditional.

In this study, we have defined a two parameter model for defining common or core microbiome membership. This definition not only facilitates a probabilistic interpretation of “common”, but also provides useful visual insights into the distribution of taxa across a set of donors (Figure S1). The two parameters of core membership are abundance and ubiquity. The “abundance” refers to the percentage of a taxon in a sample and the “ubiquity” refers to the percentage of samples that possess the taxon. As such, the ubiquity is a function of the abundance. From a probabilistic perspective, this relationship can be considered a CDF since as abundance decreases, ubiquity increases, making it monotonically increasing, continuous, and bounded at its limits between 0 and 1. However, for ease of interpretation, it is plotted as a monotonically decreasing function. Intuitively, one would expect that as the threshold of percent abundance of a taxon in question increases, fewer samples would be found in which this taxon exceeds that threshold. Thus, to define a core, it would be sufficient to identify the subset of taxa which exceed both predefined abundance and ubiquity thresholds.

**Figure 4:**
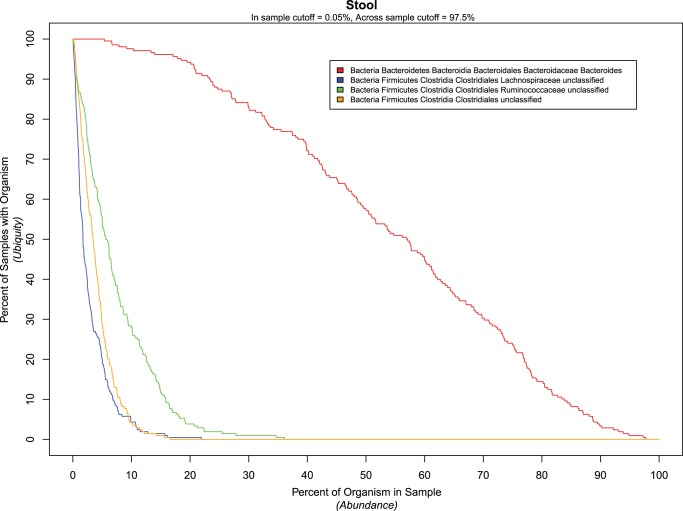
Low abundance, high ubiquity taxa. This figure helps the observer to comprehend the relationship between abundance and ubiquity when defining a core microbiome. As one would expect, increasing the abundance threshold for defining whether a sample contains a particular taxon would reduce the percentage of samples (ubiquity) that would contain it. The lines that are presented refer to all taxa in the stool samples that are in more than 97.5% of the samples with an abundance cutoff of 0.05%. The taxon *Bacteroides* (red) is both relatively highly abundant and highly ubiquitous, so its fall off is less steep than the Clostridales shown.

For example, an examination of the collection of 16 S profiles from stool (Figure 4) revealed four “core” taxa surpassing an abundance cut off of 0.05% and ubiquity cut off of 97.5%. The selection of these values serves to demonstrate the presence of low abundant taxa (found in the tail) which are simultaneously prevalent across the sample set (ubiquitous). The three taxa at low abundance are classified as members of the Class, Clostridia. Of these, two can be classified to the family level (Lachnospiraceae and Ruminococcaceae) while the classification of the third is unknown. The fourth ubiquitous taxon found at these cut offs can be classified to the genus level as *Bacteroides*. However, as evidenced by the more gradual drop off of its CDF (Figure 4), this organism would be considered part of the “common” core, since its abundance is significantly higher in a larger fraction of the total data set.

### Richness Among Individuals Predicted by Rarefaction and Curve Fitting

To estimate the richness, S_max_, of each body habitat across all individuals, a bootstrapping approach was originally used to generate a set of estimates for the number of taxonomic units discovered along the rarefaction curve. A single curve for each body habitat was derived by using the median of the discovered taxonomic units and a saturation point, S_max_, was extrapolated with curve fitting. While curve fitting this median produced compelling results, i.e. those with low mean squared errors, the key drawback to this methodology was that it did not produce CIs. These are critical for any predictive methodology, since small changes in a curve’s shape could produce large swings in S_max_. In order to compute CIs, an alternative approach was chosen to bootstrap the *sequence* of individuals multiple times, curve fit each sequence, and then generate a set of S_max_’s from which to distill the median and 95% CIs. The total number of taxonomic units encountered at a particular point of sequencing was divided by the estimated value for S_max_ to approximate the current percent coverage of population richness. This approach provides a parametric alternative to the commonly used non-parametric Good’s coverage estimator, which is simply the number of taxonomic units sampled more than once, divided by the number of units sampled in total [35].

A comparison of the fitness of four theoretical CDF curves (log-normal, gamma, Fréchet, and Pareto) using a least squares measure, indicated that for genera-based taxonomic units, the log-normal CDF was the best fit, but for the OTU-based taxonomic units, the gamma CDF was more appropriate. The wide 95% confidence intervals around the S_max_ estimates, with the upper bounds frequently a few orders of magnitude greater than the median, suggested that given the variability in donor taxonomic compositions, there was still an insufficient number of donors to produce robust results with this methodology. The variation in the shape of the rarefaction curves, as a result of only permuting the order of donors sampled, indicated that the taxonomic profiles across individuals for the same body habitat could be highly variable. This observation was corroborated by the wide 95% CIs surrounding the site medians for both the Shannon diversity and τ statistics, as discussed previously. Nonetheless, the general trend still indicated that the skin and oral samples possess a greater number of taxa (genera- and OTU-based) when compared to vaginal samples, Figure 2c and 2f subfigures. For genera-based S_max_ predictions, the skin region was statistically significantly greater than the oral region (one-sided WRST, p-value  = 0.03). This was not seen before in both taxonomic and OTU calculations for both the Shannon and τ statistic. Oral and skin regions shared the same p-values for significance indicating greater diversity when compared to the vaginal region, as seen previously. (See Tables S1a–f for full pairwise WRST comparisons of body regions).

An additional by-product of curve fitting is the ability to estimate the anticipated discovery rate of new taxonomic units upon additional sequencing. The median and 95% CIs of taxonomic discovery rates for all sampled body habitats can be found in Table 3. OTU-based results were generated using the gamma CDF while the taxonomic-based results were based on the log-normal CDF. The increase in the number of taxonomic units and the percentage of all taxonomic units, S_max_, are presented. Note that the discovery rates are a function of N, the number of donor samples collected thus far, as they are instantaneous rates for the number of donors sampled to that point. These forecasts are more dependable than S_max_, because any error from extrapolation has yet to be compounded.

**Table 3:** Expected taxonomic discover rates upon additional sampling, estimated with bootstrapping and curve fitting.

	Expected Taxonomic Units Discovered						Percent Increase of All Taxonomic Units						
	OTU			Genera			OTU			Genera			N
	Median	Lower	Upper	Median	Lower	Upper	Median	Lower	Upper	Median	Lower	Upper	
**Oral**													
Buccal mucosa	1.503	0.873	1.989	0.153	0.069	0.227	0.115	0.011	0.138	0.047	0.003	0.060	201
Hard palate	1.516	0.970	1.897	0.196	0.083	0.267	0.078	0.010	0.128	0.047	0.002	0.061	199
Keratinized gingiva	1.202	0.657	1.590	0.142	0.075	0.179	0.116	0.012	0.130	0.029	0.001	0.058	208
Palatine Tonsils	2.957	2.034	3.609	0.120	0.083	0.182	0.077	0.011	0.136	0.046	0.002	0.053	207
Saliva	2.784	1.856	3.392	0.166	0.098	0.240	0.106	0.012	0.144	0.049	0.003	0.064	183
Subgingival plaque	3.475	2.306	3.957	0.148	0.072	0.201	0.085	0.012	0.140	0.025	0.002	0.053	206
Supragingival plaque	2.930	1.910	3.865	0.130	0.073	0.189	0.057	0.011	0.136	0.024	0.001	0.052	205
Throat	2.087	1.443	2.654	0.192	0.113	0.285	0.124	0.010	0.141	0.048	0.002	0.069	198
Tongue dorsum	2.786	2.101	3.422	0.092	0.053	0.152	0.128	0.012	0.145	0.023	0.001	0.051	205
**Skin**													
Anterior nares	2.020	0.869	2.767	0.397	0.295	0.554	0.144	0.014	0.166	0.068	0.006	0.085	173
L Antecubital fossa	4.261	0.849	7.837	0.792	0.393	1.328	0.192	0.018	0.322	0.129	0.008	0.172	89
L Retroauricular crease	1.676	0.671	2.181	0.334	0.190	0.463	0.114	0.010	0.152	0.067	0.008	0.081	193
R Antecubital fossa	3.442	1.386	7.578	0.566	0.274	1.045	0.219	0.024	0.294	0.112	0.041	0.169	94
R Retroauricular crease	2.091	0.822	3.631	0.315	0.159	0.592	0.113	0.010	0.154	0.058	0.003	0.077	199
**Vaginal**													
Mid vagina	1.461	0.452	2.620	0.267	0.158	0.467	0.246	0.024	0.320	0.132	0.012	0.170	95
Posterior fornix	0.892	0.190	1.531	0.256	0.137	0.376	0.170	0.017	0.351	0.146	0.008	0.203	95
Vaginal introitus	2.080	0.637	3.507	0.252	0.115	0.498	0.231	0.025	0.368	0.129	0.005	0.171	86
**Stool**	10.502	7.608	13.401	0.104	0.060	0.146	0.143	0.013	0.158	0.044	0.002	0.053	208

For all body habitats, an additional donor sample would only lead to a fractional percentage increase in S_max_. For example, an additional donor added to the curve for stool will lead to an additional 10.5 OTUs being discovered. Due to the low diversity of the vaginal microbiome, anticipated discovery rates were similar to other body sites, even though half as many samples were collected from the donor cohort. The left and right antecubital fossae experienced lower sampling success rates, resulting in a lower number of total samples and in taxonomic discovery rates that were noticeably higher when compared to other skin sites. The calculation of these anticipated discovery rates can be used in human microbiome diversity studies as a rational aid in deciding whether additional sampling of a particular body habitat is scientifically warranted or cost effective.

### The Difference between Taxonomic-based and OTU-based Units

Detecting differences between the diversity estimated through OTU-based and genera-based taxonomic units is expected. The genera-based units are assigned to the genus level, whereas the OTU-based units are considered to be proxies for species level granularity when clustering at 97% intracluster sequence similarity. Note, however, that the 97% similarity typically associated with species is meant to be applied to a full length 16 S sequence, e.g., paired-end Sanger sequencing, not to a shorter pyrosequencing read covering only 2–3 variable regions of the 16 S gene. As a result, if a targeted variable region contains greater genetic diversity than other portions of the rDNA sequence, the 97% cut off may produce more OTUs than taxonomic-based classifications such as species, depending on the phylogeny of the bacterium. Although OTU-based taxonomic units are more sensitive to sequencing errors, they are also more likely to treat novel organisms properly and partition sequences more accurately, since they do not depend on a database of known sequences to classify against. Contrariwise, the genera-based approach will group novel sequences together as unknown, and it is common for a single known genus to represent a variable number of species.

To provide a brief comparison of a few genera-to-OTU ratios, five common genera were selected to highlight the body site specific OTU variation which underlies each genus. Table 4 contains the expected number of OTUs per 100,000 reads for the genus of *Bacteroides*, *Lactobacillus*, *Propionibacterium*, *Streptococcus*, and unknown, for the oral, skin, stool and vaginal regions. The reported ratios were normalized by sequencing depth to minimize the impact of OTUs that may have been formed due to sequencing error as read depth increases. Note that this is an over correction, as one would expect that the rate of OTU formation would be less than linear compared to the increase in read depth. Even after this normalization is applied to make the estimates more conservative, it can still be seen that when a genus is dominant within a body region, it can have an OTU composition more complex than other body regions with the same genus present. It is also noteworthy that, of the four body regions, stool had a significantly larger number of uncharacterized (unknown) organisms than the oral, skin, or vaginal regions.

**Table 4:** Expected OTUs per 100,000 reads represented for the most common genera across body regions.

Genera	Oral	Skin	Stool	Vaginal	Across All Regions
***Bacteroides***	18.39	41.17	2137.16	39.11	152.44
***Lactobacillus***	1.54	3.19	7.63	54.66	5.36
***Propionibacterium***	2.79	104.78	18.17	13.71	26.61
***Streptococcus***	33.14	7.76	9.27	15.39	20.24
**Unknown**	7.97	6.98	69.95	5.28	10.25

**Figure 5:**
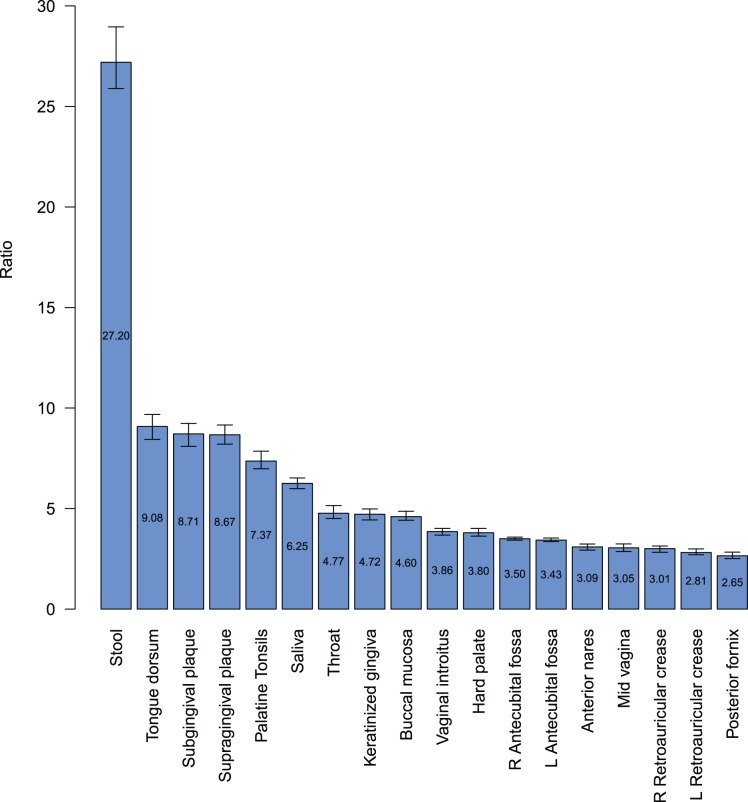
OTU-to-Genera ratios. The median ratio of OTUs to Genera was calculated and plotted from greatest to least for each body habitat. These medians and 95% confidence intervals were estimated with bootstrapping by resampling from the combined distribution of OTUs and Genera to a common read depth. The common read depth chosen was the body habitat with the least read coverage, left antecubital fossa.

Examination of the one-to-many relationships between genera and OTUs across body habitats can be visualized with the OTU-to-Genera ratio bar chart (Figure 5). Each ratio is the number of OTU-based taxonomic units divided by the number of genera-based taxonomic units. For each body habitat, OTU-based and genera-based profiles of taxonomic units were constructed using the equal-weighted combination of each donor’s profile. Because the read depth varied from body habitat to body habitat, resampling was performed to make read depths consistent. The depth of resampling used to ensure consistency was based on the left antecubital fossa, which had the shallowest coverage (328,117 reads) of all body habitats.

The OTU-to-Genera ratio varied across the various body habitats and regions indicating that the relationship between OTU-based and genera-based units is neither constant nor attributable to systematic sequencing error or classification bias alone. Although the ratio among body habitats varied, ratios were more consistent among body habitats of the same body regions. Stool samples held the greatest median OTU-to-genera ratio (27.20) while the vaginal and skin habitats revealed smaller ratios. Within the oral region, the highest median ratios were determined for the tongue dorsum (9.08), followed by the subgingival (8.71) and supragingival(8.67) plaques. These results may in part be indicative of the habitats in question as the tongue dorsum represents the papillated surface of the tongue, while the plaque samples represent biofilms adhered to a non-shedding hard surface [36]. As such, these are habitats that can generate microenvironments which favor aggregation and the long term association of diverse microbial communities.

### The Diversity of the Common versus all Taxonomic Units

The “common” taxonomic units were defined by applying a cut off of 1% abundance to the taxonomic profiles of each sample, see Figure S1. The same calculations for the Shannon diversity, τ statistic and S_max_ were performed on the filtered data and summarized in Tables 5 and 6. The proportion of all the estimated S_max_ that was considered common for the genera-based taxonomic units had a median of 0.2365 and for OTU-based taxonomic units, a median of 0.0726. These results underscore the long-tailed nature of the 16 S profiles obtained from HMP data in that most samples are dominated by a small number of taxa. This result further suggests that certainly the most abundant genera and perhaps the majority of human microbiome diversity has been captured in the HMP 16 S data sets.

**Table 5:** Diversity indices computed on “common” genera-based taxonomic units.

Location	Shannon Entropy				Tail				S_max_			N
	Median	Lower	Upper	Pooled	Median	Lower	Upper	Pooled	Median	Lower	Upper	
**Oral**												
Buccal mucosa	1.413	0.512	2.278	1.740	2.187	0.535	5.073	4.743	3,647	43	20,153	201
Hard palate	1.815	0.805	2.398	2.092	3.392	1.314	5.616	5.515	59	40	9,560	199
Keratinized gingiva	1.404	0.399	2.332	1.827	1.948	0.400	5.067	4.493	52	35	12,525	208
Palatine tonsils	2.131	1.259	2.553	2.565	4.131	1.756	6.330	7.115	67	50	8,086	207
Saliva	2.308	1.645	2.746	2.713	5.018	2.575	7.560	7.964	756	55	7,012	183
Subgingival plaque	2.343	1.609	2.783	2.924	5.116	2.592	8.043	9.364	59	47	2,090	206
Supragingival plaque	2.368	1.589	2.763	2.804	5.203	2.269	7.827	8.033	44	38	2,167	205
Throat	2.156	0.943	2.606	2.627	4.328	0.890	6.794	8.344	355	65	36,106	198
Tongue dorsum	2.095	1.386	2.488	2.476	3.811	1.920	5.619	5.682	37	30	3,487	205
**Skin**												
Anterior nares	1.301	0.398	2.095	2.046	1.623	0.427	4.292	6.096	101	52	8,922	173
L Antecubital fossa	1.499	0.229	2.672	2.481	2.783	0.300	7.660	15.626	153	100	4,651	89
L Retroauricular crease	0.670	0.000	1.978	1.210	0.667	0.000	3.747	4.955	128	57	22,355	193
R Antecubital fossa	1.472	0.208	3.009	2.475	2.323	0.305	10.141	16.991	142	112	5,444	94
R Retroauricular crease	0.677	0.000	1.876	1.282	0.671	0.000	3.561	5.186	91	62	26,787	199
**Vaginal**												
Mid vagina	0.076	0.000	1.516	0.583	0.121	0.000	1.989	2.803	39	26	8,526	95
Posterior fornix	0.000	0.000	1.437	0.464	0.000	0.000	1.914	1.478	27	20	14,278	95
Vaginal introitus	0.115	0.000	1.433	0.632	0.157	0.000	2.233	2.958	48	30	3,731	97
**Stool**	1.406	0.246	2.432	1.894	2.423	0.280	5.532	6.426	61	46	4,353	208
												

**Table 6:** Diversity indices computed on “common” OTU-based taxonomic units.

Location	Shannon Entropy				Tail				S_max_			N
	Median	Lower	Upper	Pooled	Median	Lower	Upper	Pooled	Median	Lower	Upper	
**Oral**												
Buccal mucosa	1.492	0.536	2.417	1.950	2.505	0.652	6.104	6.634	69	54	270	201
Hard palate	2.016	0.991	2.707	2.453	4.170	1.550	7.415	7.814	70	60	266	199
Keratinized gingiva	1.509	0.568	2.328	2.047	2.245	0.564	5.803	6.053	67	53	121	208
Palatine tonsils	2.360	1.466	2.888	3.126	5.307	2.219	8.844	14.954	106	92	156	207
Saliva	2.526	1.914	2.928	3.088	6.148	3.542	9.528	13.094	108	90	390	183
Subgingival plaque	2.527	1.839	2.953	3.501	6.762	3.128	10.461	22.275	126	105	159	206
Supragingival plaque	2.531	1.608	2.987	3.227	6.251	2.241	10.294	15.336	95	82	127	205
Throat	2.380	0.880	2.870	3.086	5.573	0.819	9.396	14.535	117	95	159	198
Tongue dorsum	2.240	1.532	2.745	2.761	4.636	2.325	7.674	8.262	56	50	267	205
**Skin**												
Anterior nares	1.380	0.644	2.253	2.201	1.716	0.640	4.586	8.745	102	73	682	173
L Antecubital fossa	1.636	0.149	2.714	2.629	3.292	0.192	8.878	23.104	158	130	3,886	89
L Retroauricular crease	0.654	0.000	1.865	1.209	0.647	0.000	3.987	7.623	97	70	712	193
R Antecubital fossa	1.433	0.165	2.920	2.601	2.466	0.256	10.249	25.275	159	130	233	94
R Retroauricular crease	0.646	0.000	1.913	1.306	0.646	0.000	3.834	7.720	112	77	718	199
**Vaginal**												
Mid vagina	0.078	0.000	1.786	0.631	0.122	0.000	2.638	4.184	66	35	533	95
Posterior fornix	0.000	0.000	1.750	0.529	0.000	0.000	2.452	2.302	28	20	514	95
Vaginal introitus	0.091	0.000	1.965	0.686	0.135	0.000	3.301	4.650	49	39	386	97
**Stool**	1.981	0.681	2.728	2.963	3.580	0.825	7.883	23.818	192	160	3,865	208

**Figure 6:**
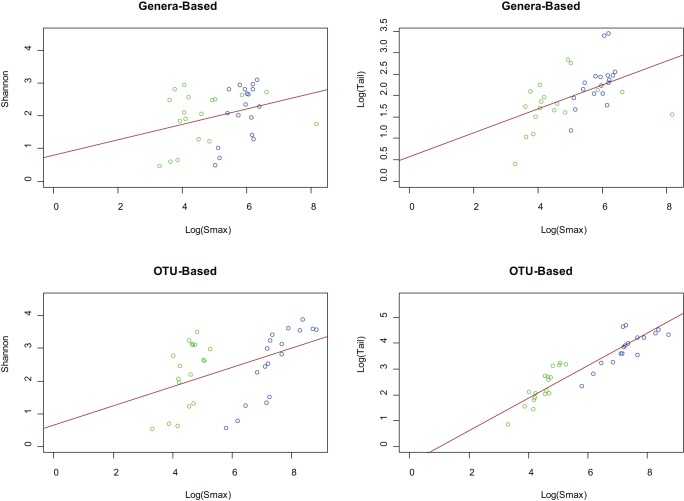
Comparison of all and “common” taxonomic units and their effect on the Shannon and τ statistics. For both genera-based and OTU-based taxonomic units, the Shannon diversity index and τ were compared against the median estimated S_max_ on all (blue) and common (green) taxonomic units. Each point in the scatterplot represents one of the 18 body habitats. There is a closer relationship between τ and S_max_ than for the Shannon diversity index, for both genera and OTU based profiles. The red line represents a simple regression line across all points.

A scatterplot of the median estimated S_max_ versus the Shannon and τ statistic computed on the pooled taxonomic profiles for both OTU-based and genera-based units on all 18 body habitats illustrates the difference of diversity estimates using only common or all taxonomic units (Figure 6.) After the application of the 1% abundance filter (green), there was not a significant change in the computed Shannon diversity indices for each body habitat when compared to all taxonomic units (blue). This result was consistent between OTU-based and genera-based units. However, an examination of the τ statistic revealed a stronger impact due to the removal of the rare taxonomic units, especially for the OTU-based taxonomic units. Computation of Spearman’s correlation coefficient between S_max_ and the Shannon diversity index resulted in the values of 0.326 (genera-based) and 0.574 (OTU-based). The higher the correlation between the diversity index and S_max_, the better the diversity index can predict the magnitude of S_max_. In contrast, examination of the τ statistic revealed a greater impact from truncating the rare taxonomic units. This is displayed by the downward shift of the τ statistic. Spearman’s correlation coefficient was measured as 0.552 and 0.941, for genera-based and OTU-based taxonomic units, respectively. These scatterplots confirm the Shannon diversity index’s inability to capture the contribution of low abundant taxa to human microbiome diversity.

### Comparing Low Abundance Diversity with the Dominance Profile

A traditional rank abundance curve is useful for determining the most dominant members of a sample, however the length of the long tails of microbiome samples and their associated low heights make them difficult to visualize. Applying a logarithmic transformation to the abundance measurements along the y-axis may ameliorate the study of the exponentially disappearing tail, however establishing a clear visual comparison between many samples is still difficult.

**Figure 7:**
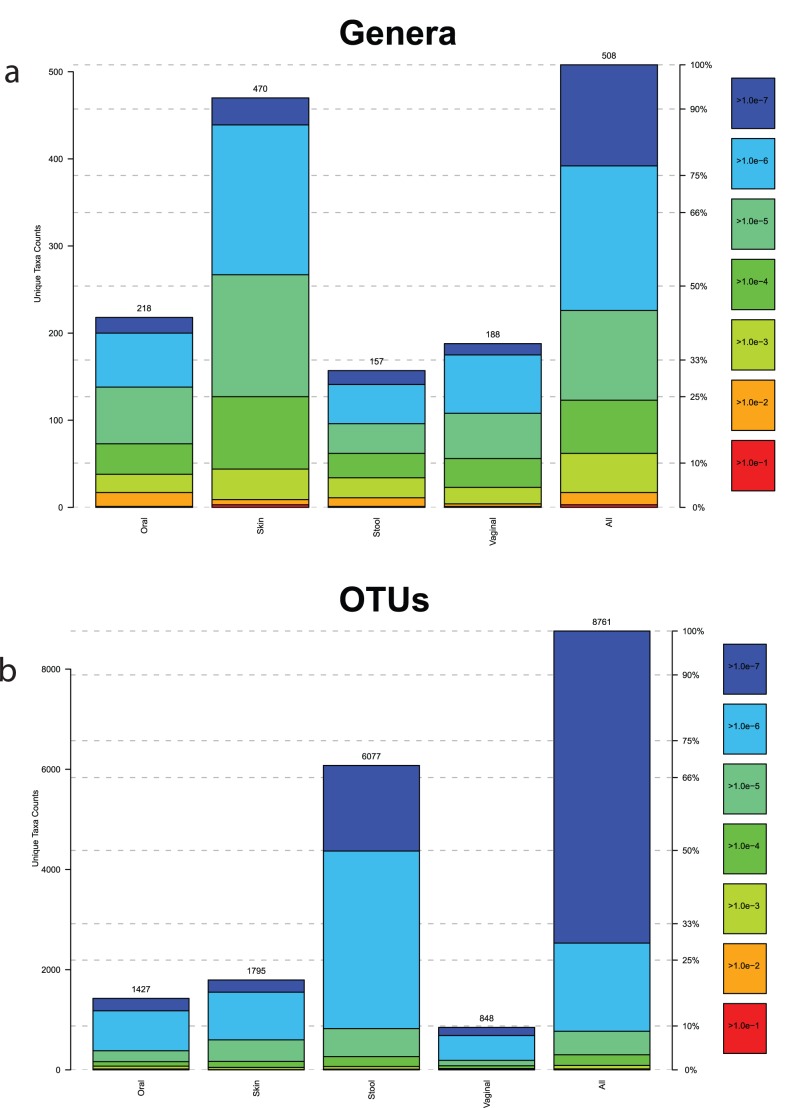
Dominance Profiles. These stacked bar plots help to compare the low abundant taxonomic units, which may be difficult to visualize with rank abundance curves alone. The number of taxonomic units for each body region is represented by the height of each bar plot. The proportions that are colored represent the relative logarithm of abundance with the color key on the left. The subpanels, a and b, represent genera and OTUs, respectively.

The dominance profile is introduced to help visualize and compare the structure and diversity contained in the long tail. It is a stacked bar plot that color codes the total taxonomic units sampled based on their logarithm abundance. The dominance profile was generated on the combined taxonomic profiles of the four body regions (Figure 7a and 7b). The height of each bar plot represents the number of taxonomic units that have been discovered in the body region and each stacked partition represents a range of that taxon’s abundance. For example, taxonomic units with a measured abundance greater than or equal to 10%, 1%, 0.1%, and 0.01%, are assigned red, orange, yellow-green and green, respectively. To make the comparison of low abundance taxonomic units comparable between samples with unequal sampling depths, an abundance filter was applied to all four body regions based on the region with the shallowest cumulative read depth (stool). This makes the lowest detectable abundance consistent among body regions.

From an examination of the results (Figures 7a and 7b), it can be seen that the majority of taxonomic units are present at a low abundance for both genera-based and OTU-based taxonomic units. For genera-based taxonomic units, the skin contains the greatest richness compared to the other body regions. However, a view of the dominance profile using OTU-based taxonomic units indicates that the stool contains the greatest richness. This confirms the unique one-to-many relationship between genera-based and OTU-based units for stool. Furthermore, these results demonstrate that the patterns of diversity discovered when comparing genera to the higher granularity units represented by OTUs, cannot be reconciled with a fixed scaling factor, or coefficient, alone. This highlights the potential value of new statistical tools, both visual and quantitative, that can be applied to resolve the patterns of taxa found in the “rare microbiosphere”.

### Conclusions

The breadth and depth of sampling provided through 16 S profiles generated by the HMP has created an unprecedented opportunity to improve assessments concerning the variability of human microbiome diversity within and between individuals and body habitats including advances to the methods and visualizations used to produce and view these assessments. Our study reveals that the patterns of diversity recovered from the HMP 16 S profiles including S_max,_ and the instantaneous rate of discovery of new taxa, differ based on the granularity of the taxonomic units (genera versus OTUs). Further, while it is possible to apply parametric methods to the estimation of maximal taxa within a body habitat using the HMP data sets, the choice of best curve fitting also varied based on taxonomic granularity.

The application of standard ecological diversity indices such as the Shannon diversity index reveals an underestimation of diversity, since much of the richness is attributed to low abundant taxa found in the long-tailed distribution of sampled taxonomic abundance. By introducing the concept of the Tail statistic, τ, we demonstrate how the analysis of the human microbiome elucidates body habitat specific characteristics which could not be detected by observing standard ecological diversity indices alone. The observation that different body habitats and individuals will have characteristic and systematic differences in both richness and evenness emphasizes the importance of quantifying these variables and employing improved methods for visualizing these results, in order to ultimately understand the nature of microbial community structure and its relationship to human health.

## Materials and Methods

### Ethics Statement

As a part of a multi-institutional collaboration, the HMP human subjects study was reviewed by the Institutional Review Boards at Baylor College of Medicine under IRB Protocol H-22895, the Washington University School of Medicine under protocol number HMP-07-001 (IRB ID# 201105198) and at the J. Craig Venter Institute under IRB Protocol Number 2008-084. All study participants gave their written informed consent before sampling and the study was conducted using the HMP Core Sampling Protocol A. Each IRB has a federal wide assurance and follows the regulations established at 45 CFR Part 46. The study was conducted in accordance with the ethical principles expressed in the Declaration of Helsinki and the requirements of applicable federal regulations.

### Computing the Tail Statistic

**Figure 8:**
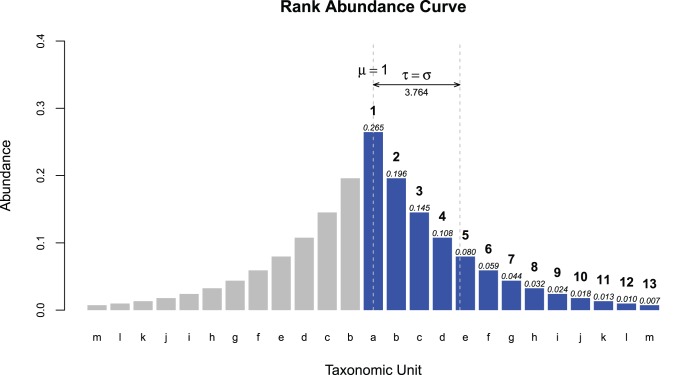
Relationship between the Tail statistic, τ, and Standard Deviation, σ. τ is the standard deviation of the rank abundance curve after reflection around the most dominant taxonomic unit, *i*  = 1. The blue bars represent the rank abundance curve. Above each bar, the probability, Pr[*i*], of the *i* ^th^ most dominant taxonomic unit has been labelled in italics. The natural numbers labelled in bold above the blue bars represent the rank, *i*, of each taxonomic unit. The name of each taxonomic unit is labelled along the x-axis. The grey bars represent the mirror image of the rank abundance curve. Treating *i*  = 1of the symmetric distribution as μ  = 1, the standard deviation, σ, is then 3.764, which also represents τ for this rank abundance curve and sample.

The Tail statistic is essentially the standard deviation of the rank abundance curve had it been made symmetric by reflection around the most abundant taxon, i  = 1. The Tail statistic, τ, is defined as:
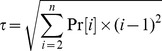

*n* is the number of taxa discovered in the sample. Pr[*i*] is the proportion of the i^th^ most abundant taxa. The sum of Pr[*i*] from *i*  = 1 to *n*, should equal 1, where Pr[*i*] ≥Pr[*i+1*], for all *i*. Unlike Hill’s numbers, such as the Shannon or Simpson’s diversity indices where the impact of low abundance taxonomic units are dampened by the power Pr[i] is raised to, τ measures the dispersion of the ranked taxonomic abundances away from the most abundance taxonomic unit. The value of 1 is subtracted from each *i*, because 1 represents the mean of the rank abundance curve had it been made into a symmetric distribution by reflection. See Figure 8 for a visualization of how the τ statistic is related to the standard deviation. From the values in Figure 8, the following is a sample calculation of τ, for that rank abundance curve.



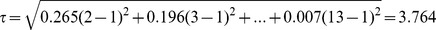



One of the characteristics that can be exploited by the Tail statistic’s relationship to standard deviation is Chebyshev’s inequality. Chebyshev’s inequality provides a lower bound for the proportion of the population that can be found within *k* standard deviations, σ, of the mean, μ, independent of the distribution type. Since the scale of τ is the same as that of the standard deviation, Chebyshev’s inequality can also be applied to estimate the number of taxa necessary to capture a defined percentage of the population. The τ-modified Chebyshev’s Inequality is:
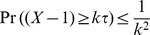
X is the random variable which represents the measured number of taxa in the sample. To estimate the number of taxa necessary to capture >95% of the population, τ is multiplied by *k*  = 4.4725, then added to 1. To capture >99% of the population, τ would be multiplied by 10 and then added to 1. Alternatively, by counting up the number of number of taxonomic units, *D*, seen in a sample, and using a previously estimated τ, provides a lower bound on the proportion of taxonomic units discovered so far.



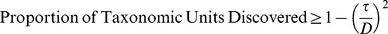



### Computing the Rarefaction Curve

Each rarefaction curve was calculated by bootstrapping the donors and the reads from the taxonomic distribution of each donor, based on either the genera-based or OTU-based taxonomic unit distributions for each donor and body habitat.

To compute the median and confidence intervals of species richness, S_max_, each bootstrapped rarefaction curve was curve fit to estimate its saturation point. Each bootstrapped rarefaction curve thus represented an alternative sequence ordering of donors being selected with replacement from the donor pool. This was important because the variability in each individual’s body habitat species richness and evenness could significantly modulate the shape of the rarefaction curve depending on the order of when each donor was encountered.

Let us define the number of discovered taxonomic units, *t(n)*, (y-axis) for each donor count, *n,* (x-axis). For example, if a 3^rd^ donor (n  = 3) had been sequenced, the number of taxonomic units discovered among the 3 donors might be 200, so *t*(3)  = 200, where *t*(*n*) ≤ *t*(*n* +1). Let us define the *n* ^th^ donor, *d_n_*, who is a member of all available donors, *D*, and a taxonomic profile, *p(d_n_)*, as the distribution of taxonomic units from *d_n_*’s body site. Choose a donor, *d_n_* randomly with replacement from *D*. Then for this additional donor, *d_n_*, sample *r* reads from *p*(*d_n_*). *t*(*n*) is then calculated by counting up all the unique taxonomic units that have been discovered among *d_i_* where *i*  = 1, 2, … *n* by looking at the *r×n* reads.

### Curve Fitting the Rarefaction Curve

The rarefaction curve can be thought of as a CDF, if S_max_, the maximum number of taxonomic units across the population, is used as a normalization factor to convert the number of discovered taxonomic units to be within the range of 0 and 1. The CDFs of four commonly used theoretical probability distributions (log-normal, gamma, Pareto, and Fréchet), were fit to each rarefaction curve, see Figure S2. Fitting of the rarefaction curve to the same probability density function (PDF) was not attempted, because the derivative of the rarefaction curve produced a very unsmooth result due to the variability of donor diversity. To fit the theoretical CDF to the targeted rarefaction curve required the optimization of each distribution’s parameters, usually the shape and scale. Any location parameters were generally fixed at zero, because the rarefaction curve starts at zero donors and zero discovered taxonomic units. In addition to the standard distribution specific parameters, the additional parameter S_max_ was also included for optimization. The cost, or objective, function to be minimized was the root mean square distance (RMSD) between the rarefaction curve and the theoretical parameterized CDF multiplied by S_max_. The RMSD was chosen because it allowed for the comparison of fitness between curve fits of unequal donor size. The R function, optim, was used with the Nelder-Mead algorithm to perform the optimization. To avoid the common problem with stopping at local minima, multiple optimization runs were performed. An initial run was performed with S_max_ seeded at the number of taxa in the target sample’s distribution. After a first S_max_ estimate was made, a new set of optimizations was seeded from various distances relative to the first S_max_ estimate. The set of parameters with the lowest RMSD value across all seeded optimizations was reported.

### Estimating Taxonomic Discovery Rates

The taxonomic discovery rates were estimated by using the probability distribution function (PDF) of the CDF utilized in the fitted curve. The log-normal and gamma distribution were selected for the genera-based and OTU-based taxonomic units, respectively. Since the PDF is the first derivative of the CDF, an instantaneous discovery rate can be computed with the PDF simply as a function of N (samples collected so far) because the other parameters of the distribution function, i.e., shape and scale, have already been estimated by curve fitting. The median and 95% CIs of the rates were estimated using the same bootstrapped rarefaction curves used to estimate the median and 95% CIs for S_max_.

### Description of Donor Recruitment, Sampling and 16 S rRNA Gene Sequencing

Subjects were recruited, enrolled and sampled at two recruitment centers (Baylor College of Medicine, Houston, TX and Washington University, St. Louis, MO) as detailed in (Aagaard K, Petrosino J, Keitel W, Watson M, Katancik J, et al. “A Comprehensive Strategy for Sampling the Human Microbiome,” In preparation.). In brief, subjects between the ages of 18 and 40 years who passed a screening for systemic health based on oral, cutaneous, and body mass exclusion criteria (see http://hmpdacc.org/micro_analysis/microbiome_sampling.php) were eligible for enrollment and subsequently approved by the Institutional Review Boards of the respective recruitment centers. Samples were obtained from 15 male or 18 female body sites (habitats) using a common sampling protocol (see http://hmpdacc.org/doc/HMP_Clinical_Protocol.pdf). The following body habitats were sampled: nine oral samples (saliva, swabs from the buccal mucosa, tongue, keratinized gingiva, hard palate, tonsils, and throat, and sub- and supragingival plaque scraping); three vaginal samples (swabs from the vaginal introitus, posterior fornix, and vaginal midpoint); four skin samples (bilateral retroauricular crease and antecubital fossa swabs); airways (both anterior nares, swabbed and pooled); and stool (self collected by commode kit). Genomic DNA from all samples was isolated using the Mo Bio PowerSoil DNA Isolation Kit (http://www.mobio.com) as detailed in (Aagaard K, Petrosino J, Keitel W, Watson M, Katancik J, et al. “A Comprehensive Strategy for Sampling the Human Microbiome,” submitted, and The Human Microbiome consortium “Mapping the human microbiota: resources from the Human Microbiome Project,” in preparation, see also http://hmpdacc.org/doc/sops_2/manual_of_procedures_v11.pdf), and subsequently amplified and sequenced on the Roche-454 FLX Titanium platform according to the “HMP 16 S Protocol” (Jumpstart Consortium Human Microbiome Project Data Generation Working Group, “High-throughput methods for 16 S sequencing in human metagenomics,” submitted, and see also (http://www.hmpdacc.org/).

### 16S rRNA Sequence Data Processing and Data Set

Sequence reads were processed using a pipeline constructed by the HMP Consortium as described in (The Human Microbiome Consortium, “Structure, Function and Diversity of the Human Microbiome in an Adult Reference Population,” submitted.). Data from the “high stringency” pipeline was used in this analysis and quality was assured with the following steps described here in brief. For sample multiplex barcode deconvolution and 16 S primer trimming, a one nucleotide unambiguous mismatch to the sample barcode and up to two nucleotide mismatches to the adjacent PCR primer were allowed, respectively. Sequences with an ambiguous base call or a homopolymer stretch longer than eight nucleotides were removed from subsequent analyses. The high stringency pipeline incorporated a strategy of calculating the average quality score within a 50 nucleotide window that was shifted along the sequence. When the average quality score of the window decreased to <35, the sequence was trimmed. After trimming, all sequences were aligned using a NAST-based sequence aligner [37] to a custom reference based on the SILVA [38] database of curated alignments. Sequences shorter than 200 nucleotides or that did not align to the anticipated region of the reference alignment were removed and extraneous bases that extended beyond the targeted variable region were trimmed. Chimeric sequences were then identified using the Mothur implementation of the ChimeraSlayer algorithm which was trained to the “Gold” database (http://microbiomeutil.sourceforge.net). These filtered alignments formed the data set used for the generation of taxonomic classifications using RDP [22].

From the read processing described above, a total of 3,044 samples (17,371,356 total reads) were analysed. The breakdown of sample and sequencing statistics by body habitat is given in Table S3.

## Supporting Information

Figure S1 **Idealized rank abundance curve with common and rare taxa labelled.** The common taxa have been defined as any taxa with abundance greater than 1% (blue). The rare taxonomic units tend to outnumber the common taxonomic units although their proportion of abundance is low.
(EPS)

Figure S2 **Bootstrapped instance of rarefaction curve and resultant fitted curves.** This is an example of a resultant curve fit for keratinized gingiva using OTUs for a single instance of bootstrapping. Bootstrapping the sequence of donors was necessary to generate a median and 95% confidence intervals for S_max,_ for each body site. From this methodology, it is possible to compare various models by looking at the Root Mean Square Deviation (RMSD) outcomes. The smaller the RMSD, the better the fit.
(EPS)

Table S1 **Wilcoxon Rank Sum Test P-values for body region differences.** The null hypothesis is row body regions are less than or equal to the column body regions. The alternative hypothesis is that the row body regions are greater than the column body regions. For example, looking at genera-based taxonomic units for the Shannon diversity, the oral body region had significantly greater diversity than the vaginal body region, p-value  = 0.0045 with an α of 0.05. Statistically significant differences are highlighted in blue. P-values were not corrected for multiple comparisons.
(DOC)

Table S2 **Mapping of body habitat to abbreviations.**
(DOC)

Table S3 **Sampling and read depths across body habitats.**
(DOC)
