# Supplementary material for: Analyses of the Microbial Diversity across the Human Microbiome
Source: PLoS One. 2012 Jun 13;7(6):e32118. doi: 10.1371/journal.pone.0032118 (PMC3374608; doi:10.1371/journal.pone.0032118)
Supplement: Table S1 — Wilcoxon Rank Sum Test P-values for body region differences. The null hypothesis is row body regions are less than or equal to the column body regions. The alternative hypothesis is that the row body regions are greater than the column body regions. For example, looking at genera-based taxonomic units for the Shannon diversity, the oral body region had significantly greater diversity than the vaginal body region, p-value = 0.0045 with an α of 0.05. Statistically significant differences are highlighted in blue. P-values were not corrected for multiple comparisons. (DOC) [file pone.0032118.s003.doc]

|  | **Table S1a. Genera: Shannon** | | | |  |  | **Table S1b. OTU: Shannon** | | | |
| --- | --- | --- | --- | --- | --- | --- | --- | --- | --- | --- |
|  | **Oral** | **Skin** | **Vaginal** | **Stool** |  |  | **Oral** | **Skin** | **Vaginal** | **Stool** |
| **Oral** | 0.5177 | **0.0095** | **0.0045** | 0.2000 |  | **Oral** | 0.5177 | **0.0095** | **0.0045** | 0.4000 |
| **Skin** | 0.9940 | 0.5422 | **0.0179** | 0.6667 |  | **Skin** | 0.9940 | 0.5422 | **0.0179** | 1.0000 |
| **Vaginal** | 1.0000 | 1.0000 | 0.5903 | 1.0000 |  | **Vaginal** | 1.0000 | 1.0000 | 0.5903 | 1.0000 |
| **Stool** | 0.9000 | 0.5000 | 0.2500 | 1.0000 |  | **Stool** | 0.7000 | 0.1667 | 0.2500 | 1.0000 |
|  |  |  |  |  |  |  |  |  |  |  |
|  | **Table S1c. Genera: Tail** | | | |  |  | **Table S1d. OTU: Tail** | | | |
|  | **Oral** | **Skin** | **Vaginal** | **Stool** |  |  | **Oral** | **Skin** | **Vaginal** | **Stool** |
| **Oral** | 0.5177 | 0.3497 | **0.0045** | 0.2000 |  | **Oral** | 0.5177 | 0.0734 | **0.0045** | 1.0000 |
| **Skin** | 0.6968 | 0.5422 | **0.0179** | 0.6667 |  | **Skin** | 0.9441 | 0.5422 | **0.0179** | 1.0000 |
| **Vaginal** | 1.0000 | 1.0000 | 0.5903 | 1.0000 |  | **Vaginal** | 1.0000 | 1.0000 | 0.5903 | 1.0000 |
| **Stool** | 0.9000 | 0.5000 | 0.2500 | 1.0000 |  | **Stool** | 0.1000 | 0.1667 | 0.2500 | 1.0000 |
|  |  |  |  |  |  |  |  |  |  |  |
|  | **Table S1e. Genera: Smax** | | | |  |  | **Table S1f. OTU: Smax** | | | |
|  | **Oral** | **Skin** | **Vaginal** | **Stool** |  |  | **Oral** | **Skin** | **Vaginal** | **Stool** |
| **Oral** | 0.5177 | 0.9790 | **0.0045** | 0.1000 |  | **Oral** | 0.5177 | 0.0559 | **0.0045** | 1.0000 |
| **Skin** | **0.0300** | 0.5422 | **0.0179** | 0.1667 |  | **Skin** | 0.9585 | 0.5422 | **0.0179** | 1.0000 |
| **Vaginal** | 1.0000 | 1.0000 | 0.5903 | 1.0000 |  | **Vaginal** | 1.0000 | 1.0000 | 0.5903 | 1.0000 |
| **Stool** | 1.0000 | 1.0000 | 0.2500 | 1.0000 |  | **Stool** | 0.1000 | 0.1667 | 0.2500 | 1.0000 |

**Tables S1a-f. Wilcoxon Rank Sum Test P-values for body region differences.**
