# Supplementary material for: Analyses of the Microbial Diversity across the Human Microbiome
Source: PLoS One. 2012 Jun 13;7(6):e32118. doi: 10.1371/journal.pone.0032118 (PMC3374608; doi:10.1371/journal.pone.0032118)
Supplement: Table S2 — Mapping of body habitat to abbreviations. (DOC) [file pone.0032118.s004.doc]

| **Body Habitat** | **Abbreviation** |
| --- | --- |
| **Oral** |  |
| Buccal mucosa | BM |
| Hard palate | HP |
| Keratinized gingiva | KG |
| Palatine tonsils | PT |
| Saliva | SLV |
| Subgingival plaque | SBP |
| Supragingival plaque | SPP |
| Throat | TH |
| Tongue dorsum | TD |
| **Skin** |  |
| Anterior nares | AN |
| L Antecubital fossa | LAF |
| L Retroauricular crease | LRC |
| R Antecubital fossa | RAF |
| R Retroauricular crease | RRC |
| **Vaginal** |  |
| Mid vagina | MV |
| Posterior fornix | PF |
| Vaginal introitus | VI |
| Stool | STL |

**Table S2. Mapping of body habitat to abbreviations.**
