# Supplementary material for: Analyses of the Microbial Diversity across the Human Microbiome
Source: PLoS One. 2012 Jun 13;7(6):e32118. doi: 10.1371/journal.pone.0032118 (PMC3374608; doi:10.1371/journal.pone.0032118)
Supplement: Table S3 — Sampling and read depths across body habitats. (DOC) [file pone.0032118.s005.doc]

| **Body Habitat** |  | **Num Donors** | **Num Reads** | **Median Reads/Donor** | **Mean Reads/Donor** |
| --- | --- | --- | --- | --- | --- |
| **Oral** |  |  |  |  |  |
|  | Buccal mucosa | 201 | 1,137,747 | 4,506 | 5,660 |
|  | Hard palate | 199 | 1,074,137 | 4,627 | 5,398 |
|  | Keratinized gingiva | 208 | 1,120,177 | 4,409 | 5,385 |
|  | Palatine Tonsils | 207 | 1,191,035 | 5,193 | 5,754 |
|  | Saliva | 183 | 937,282 | 4,304 | 5,122 |
|  | Subgingival plaque | 206 | 1,210,342 | 5,145 | 5,875 |
|  | Supragingival plaque | 205 | 1,250,361 | 5,275 | 6,099 |
|  | Throat | 198 | 1,086,788 | 4,822 | 5,489 |
|  | Tongue dorsum | 205 | 1,263,488 | 5,539 | 6,163 |
| **Skin** |  |  |  |  |  |
|  | Anterior nares | 173 | 810,000 | 4,028 | 4,682 |
|  | L Antecubital fossa | 89 | 328,117 | 2,869 | 3,687 |
|  | L Retroauricular crease | 193 | 1,090,726 | 4,348 | 5,651 |
|  | R Antecubital fossa | 94 | 393,115 | 2,571 | 4,182 |
|  | R Retroauricular crease | 199 | 1,158,715 | 4,545 | 5,823 |
| **Vaginal** |  |  |  |  |  |
|  | Mid vagina | 95 | 638,614 | 5,514 | 6,722 |
|  | Posterior fornix | 95 | 671,623 | 5,561 | 7,070 |
|  | Vaginal introitus | 86 | 519,057 | 5,545 | 6,036 |
| **Stool** |  | 208 | 1,490,032 | 6,233 | 7,164 |
| **Total** |  | 3,044 | 17,371,356 |  | 5,707 |

**Table S3. Sampling and read depths across body habitats.**
